# Aflibercept Plus FOLFIRI as Second-Line Treatment for Metastatic Colorectal Cancer: A Single-Institution Real-Life Experience

**DOI:** 10.3390/cancers13153863

**Published:** 2021-07-31

**Authors:** Daniele Lavacchi, Giandomenico Roviello, Elisa Giommoni, Lorenzo Dreoni, Silvia Derio, Marco Brugia, Amedeo Amedei, Serena Pillozzi, Lorenzo Antonuzzo

**Affiliations:** 1 Clinical Oncology Unit, Careggi University Hospital, 50134 Florence, Italy; lavacchid@aou-careggi.toscana.it (D.L.); giommonie@aou-careggi.toscana.it (E.G.); lorenzodreoni@gmail.com (L.D.); silviaderio@yahoo.com (S.D.); brugiamarco@gmail.com (M.B.); serena.pillozzi@unifi.it (S.P.); 2 Department of Health Science, University of Florence, 50134 Florence, Italy; giandomenico.roviello@unifi.it; 3 Department of Experimental and Clinical Medicine, University of Florence, 50134 Florence, Italy; amedeo.amedei@unifi.it

**Keywords:** aflibercept, colorectal cancer, safety, prognostic factors, bone metastasis

## Abstract

**Simple Summary:**

The continuum of care for mCRC might include anti-angiogenic drug as anti-VEGF/VEGFR moAb and recombinant proteins in combination with fluoropyrimidine-based regimens in first- and second-line treatment, and multikinase inhibitors in refractory patients. The addition of aflibercept to FOLFIRI has been demonstrated to improve survival in patients with metastatic colorectal cancer (mCRC) who progressed after receiving a standard oxaliplatin-based regimen. In this retrospective, single-institution, observational study we collected clinical data from mCRC patients who received aflibercept in combination with FOLFIRI in routine clinical practice to describe feasibility and efficacy of this regimen in a real-world population. Aflibercept-FOLFIRI is a feasible second-line treatment for mCRC in a real-life setting, and PFS in first-line therapy >12 months resulted as the only predictive marker of better survival.

**Abstract:**

The addition of aflibercept to FOLFIRI has been demonstrated to improve survival in patients with metastatic colorectal cancer (mCRC) who progressed after receiving a standard oxaliplatin-based regimen. In this retrospective, single-institution, observational study we collected clinical data from mCRC patients who received aflibercept in combination with FOLFIRI in routine clinical practice from October 2012 to March 2021 to describe feasibility and efficacy of this regimen in a real-world population. Forty-nine patients receiving aflibercept-FOLFIRI as second-line treatment were identified, 40.8% of whom were aged over 65 years. The majority of patients had multi-organ metastases (73.5%), and had previously received bevacizumab in combination with chemotherapy (CT) as first-line treatment (79.6%). Median overall survival (OS) and progression-free survival (PFS) were 13 and 6 months, respectively; overall response rate (ORR) and disease control rate (DCR) were 12.3% and 49.1%, respectively. Several factors were associated with survival in univariate analysis, including PFS in first-line therapy, number of metastatic sites, bone metastases and others. However, in multivariate analysis, only PFS in first-line CT over 12 months was significantly associated with better OS (HR 0.32; 95% CI 0.13–0.79; *p* = 0.01). Hypertension was the most commonly reported grade (G) 3–4 adverse event (AE), affecting 18.4% of the overall population. Thromboembolic events were observed in 16.3% of patients, hemorrhagic events in 10.2%, and proteinuria in 8.2%. Neutropenia was the most frequently observed hematological G3–4 AE with an incidence of 10.2%. Aflibercept-FOLFIRI has been confirmed as a feasible second-line treatment for mCRC in a re-al-life setting, and PFS in first-line therapy >12 months resulted as the only predictive marker of better survival.

## 1. Introduction

Colorectal cancer (CRC) is the third most commonly diagnosed cancer worldwide, with approximately 1.9 million new cases and 935,000 deaths annually [1].

Since angiogenesis was identified as a fundamental process for tumor development and growth, from the early pathogenesis to clinically evident disease, it was included among the hallmarks of cancer. Angiogenesis is a complex process by which new blood vessels are formed from endothelial precursor, mediated through several ligands and receptors that work in tight regulation [2]. A group of glycoproteins, including the the vascular endothelial growth factors (VEGFs) (VEGF-A, VEGF-B, VEGF-C, and VEGF-D) and the placental growth factor (PIGF), act as effectors of angiogenesis interacting with VEGF receptors and neuropilin co-receptors [3]. In addition, there are multiple isoforms of VEGF-A (VEGF_121_, VEGF_165_, VEGF_189_, and VEGF_206_). A crosstalk between the VEGF signaling pathway and other angiogenic signaling pathways such as the angiopoietins (Ang-1, Ang-2, and Ang-4) and the Notch receptors (Notch 1 and Notch 4) and their ligands occurs. Furthermore, integrins and hypoxia (through HIF-1α and HIF-2α) may affect VEGF and other signaling components in tumor angiogenesis [4]. Antiangiogenic therapy constitutes a cornerstone of the treatment of patients with metastatic CRC (mCRC) [5,6,7]. In the last three decades, monoclonal antibodies (moAbs) against the VEGF/VEGF receptor (VEGFR) pathway and anti-angiogenic multikinase inhibitors have been widely used in the treatment of mCRC, both in the first and subsequent lines of therapy [5,6,7,8,9,10,11,12].

Aflibercept is a fusion protein that binds VEGFA, VEGFB, and PlGF. Acting as a soluble receptor with a higher affinity than the endogenous receptors, it prevents the activation of the angiogenesis [12].

In August 2012, the US Food and Drug Administration (FDA) approved aflibercept in combination with FOLFIRI for the treatment of patients with mCRC who progressed after receiving a standard oxaliplatin-based regimen. The approval was based on the results of the randomized phase III VELOUR trial which showed the superiority of the combination of aflibercept with FOLFIRI over placebo with FOLFIRI in terms of overall survival (OS) (mOS 13.5 vs. 12.1 months, respectively; HR 0.817; 95.34% CI, 0.713–0.937, *p* = 0.0032), progression-free survival (PFS) (mPFS 6.9 vs. 4.7 months, respectively; HR 0.758; 95% CI, 0.661–0.869, *p* < 0.0001), and overall response rate (ORR) (ORR 19.8% (95% CI, 16.4–23.2%) vs. 11.1% (95% CI, 8.5–13.8%), respectively; *p* = 0.0001). As expected, patients treated with aflibercept reported a higher rate of grade (G) 3–4 adverse events (AEs) (83.5%) compared to those treated with placebo (62.5%). In particular, G3–4 hypertension was observed in 19.3% vs. 1.5%, diarrhea in 19.3% vs. 7.8%, neutropenia in 36.7% vs. 29.5% hemorrhage in 2.9% vs. 1.7%, arterial thromboembolic events in 1.8% vs. 0.5%, and venous thromboembolic events in 7.9% vs. 6.3% [12].

To date, efficacy and safety of aflibercept plus FOLFIRI in routine clinical practice have been explored in several observational studies and early access programs (Table 1). Overall, the safety profile and real-world treatment feasibility (i.e., incidence of AEs, treatment exposure, anti-VEGF class events, etc.) may differ from the pivotal trials and, as a consequence, the achievement of clinical outcomes may not be optimal.

The purpose of this observational retrospective study was to evaluate the feasibility of aflibercept plus FOLFIRI in a real-world, unselected population, with a series of unfavorable clinical prognostic factors, potentially underrepresented in clinical trials. As additional analysis, this study explored the role of prognostic markers to identify potential subgroups of patients characterized by longer survival.

## 2. Patients and Methods

### 2.1. Study Population

In this single-institution, retrospective, observational study, we collected clinical data from patients who received aflibercept in combination with FOLFIRI in routine clinical practice at Clinical Oncology Unit, Careggi University Hospital (Florence, Italy), from October 2012 to March 2021.

The study population consisted of patients with histologically confirmed diagnosis of mCRC who progressed after a first-line oxaliplatin-based chemotherapy (CT). Other inclusion criteria were: age ≥ 18 years and written informed consent. Patients who received aflibercept for other indications were excluded. The study was conducted in accordance with the Declaration of Helsinki. The protocol was approved by the Comitato Etico Regionale for clinical experimentation of Toscana region (Italy), Area Vasta Centro section, number: 17332_oss.

### 2.2. Treatment

All patients received at least one cycle of aflibercept-FOLFIRI regimen, which consisted of aflibercept 4 mg/kg on day 1, irinotecan 180 mg/m^2^ on day 1, levofolinate 200 mg/m^2^ on day 1, and 5-fluorouracil 400 mg/m^2^ bolus injection followed by 48 h continuous infusion of 5-fluorouracil 2400 mg/m^2^ on day 1. All drugs were administered intravenously every two weeks. Given the observational nature of the study, dose modifications, delays, treatment discontinuations, and premedications were performed as per clinical practice.

### 2.3. Data Collection and Study Endpoints

Clinical data including all available demographic information, medical history, diagnosis, treatment, pathological results, molecular analyses (including RAS, BRAF and microsatellite instability (MSI) status), clinical outcomes, AEs, and laboratory alterations were collected from patients’ medical records. AEs were graded according to the National Cancer Institute Common Terminology Criteria for AEs version 4.03 [24] Tumor response evaluation was performed at least every 3 months, or before if clinically indicated by a chest-abdomen computed tomography. Radiological response was assessed according to RECIST, version 1.1 [25]. Disease progression was assessed as either radiological or clinical progression.

The primary endpoint was the evaluation of the efficacy in terms of OS. Secondary endpoints included PFS, ORR, disease control rate (DCR), safety, and the evaluation of treatment adherence and potential prognostic factors.

OS was defined as the time from the start of treatment to death from any cause. PFS was defined as the time from the beginning of the treatment to disease progression or death from any cause.

### 2.4. Statistical Analysis

Treatment adherence, safety, and correlation of clinical, biological, pathological factors and survival outcomes were analyzed. Statistical comparisons for categorical variables were performed using χ^2^ test. Time-to-event endpoints were estimated using the Kaplan-Meier method. Survival distributions for specific subgroups of patients were tested with a log-rank test. A *p*-value of 0.05 or lower was considered to be statistically significant. Parameters with a statistically significant log-rank test were considered independent variables and included in the multivariate Cox proportional hazard regression linear model to compare hazard ratio (HR) and 95% confidence interval (95% CI). All analyses were performed using the STATA, version 12.0; StataCorp LLC, College Station TX, USA.

## 3. Results

### 3.1. Patient Characteristics

From October 2012 to March 2021, 49 patients with mCRC treated with aflibercept-FOLFIRI as second-line treatment were identified. Patients’ characteristics are described in Table 2. Twenty-seven patients (55.1%) were female and 22 (44.9%) were male; 40.8% of patients (*n* = 20) were aged over 65 years. Eastern Cooperative Oncology Group performance status (ECOG PS) was 0 in 77.6% of patients and 1 in 22.4%. The prevalent primary tumor location was left (67.3%); 18 patients (36.7%) had a mucinous component at the histological evaluation. Thirty-six patients (73.5%) had multi-organ metastases, including liver (73.5%), lung (51%), lymph nodes (49%), and peritoneum (26.5%). KRAS mutation was detected in 34 patients (69.4%) and NRAS mutation in three (6.1%); BRAF mutation was found in two out of 41 samples analyzed (4.9%). Among patients with MSI data available (*n* = 18), 11.1% had MSI-high tumor. The majority of patients (89.8%) had a weight loss of more than five kilograms in the last three months.

Thirty-four patients (69.4%) had received previous surgery of the primary tumor and nine (18.4%) had received adjuvant CT. The most frequent best response to first-line CT was partial response (PR) in 40.8% and stable disease (SD) in 24.5%, with 6-month PFS and 12-month PFS rates of 77.6% and 35.9%, respectively. The majority of patients (79.6%) had received bevacizumab in combination with CT as first-line treatment (Table 3).

Among cardiovascular comorbidities, prior arterial hypertension was reported in 16 patients (41%), and thromboembolic or hemorrhagic events in six (12.2%) (Table 4).

### 3.2. Treatment

During the study, patients received a median of seven cycles of treatment (range 1–13) and 17 patients (43.7%) more than 10 cycles. CT and aflibercept dose reduction occurred in 35 (71.4%) and 13 (26.5%) patients, respectively. Twenty patients (40.8%) required treatment interruption (FOLFIRI, aflibercept or both) due to AEs. Thirty-one patients (63.3%) received further lines of treatment: trifluridine/tipiracil (*n* = 10), regorafenib (*n* = 10), and other regimens in 11 patients (Table 5).

### 3.3. Efficacy

**Figure 1:**
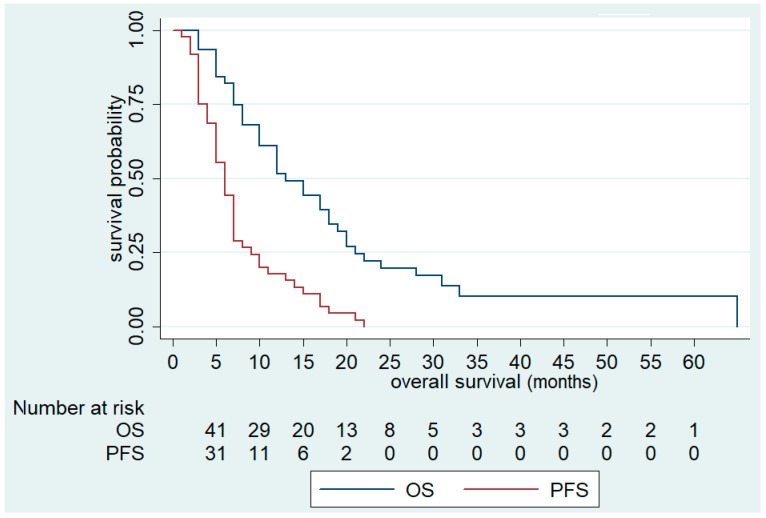
Overall Survival (OS) and Progression-Free Survival (PFS) of FOLFIRI+aflibercept in the overall population.

Overall, the median OS was 13 months (95% CI; 10–18) and median PFS was 6 months (95 % CI 5–7) (Figure 1). Complete response (CR), PR, and SD were recorded in 2 (4.1%), 4 (8.2%) and 18 (36.7%) patients, respectively, yielding an ORR and DCR of 12.3% and 49.1%, respectively. Response was not evaluable for three patients (6.1%) (Table 5).

**Figure 2:**
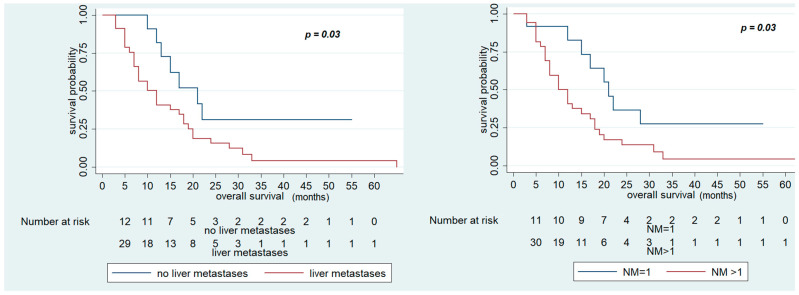
Overall Survival (OS) of FOLFIRI+ aflibercept according to the presence of liver metastases and number of metastatic sites.

Univariate analysis showed that ECOG PS 1, number of metastatic sites >1, liver metastases, and bone metastases were negatively associated with OS. In fact, OS was significantly reduced in patients with ECOG PS 1 (10 (95% CI; 3–12) vs. 17 months (95% CI 10–20), *p* = 0.03), more than one metastatic site involved (12 (95% CI 8–17) vs. 21 months (95% CI 12–not reached), *p* = 0.03), liver metastases (12 (95% CI 7–18) vs. 21 months (95% CI 12–not reached), *p* = 0.03), and bone metastases (3 (95% CI 3–not reached) vs. 15 months (95% CI 10–19), *p* < 0.01) (Table 6, Figure 2, Figures S1 and S2).

**Figure 3:**
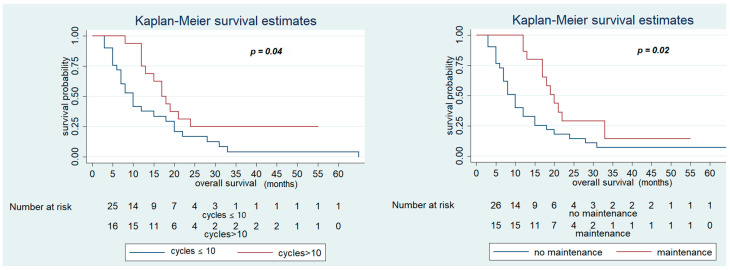
Overall Survival of FOLFIRI+ aflibercept according to the maintenance treatment and number of cycles of therapy > 10.

**Figure 4:**
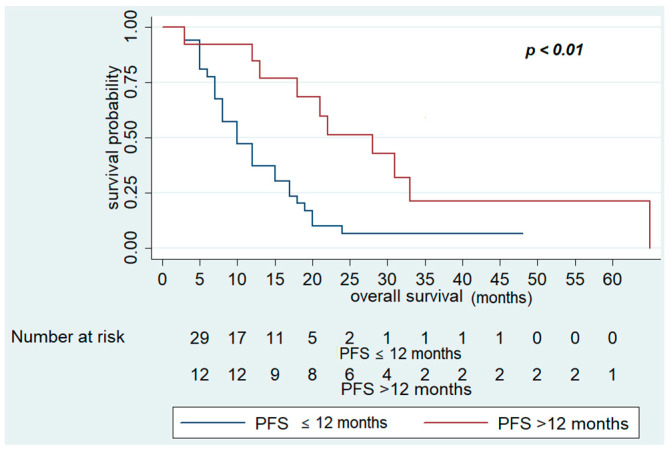
Overall Survival of FOLFIRI+ aflibercept according to PFS in first-line chemotherapy >12 months.

In contrast, OS was significantly improved in patients who had PFS in first-line CT > 12 months (28 (95% CI 13–33) vs. 12 months (95% CI 7–15), *p* < 0.01), who received maintenance treatment with aflibercept (20 (95% CI 13–33) vs. 12 months (95% CI 7–15), *p* = 0.02), and number of cycles of aflibercept-FOLFIRI >10 (17 (95% CI 12–2)) vs. 12 months (95% CI 7–18), *p* = 0.04) (Figure 3 and Figure 4). Other variables, including primary site (*p* = 0.3) and RAS status (*p* = 0.7), did not show statistically significant effects on survival (Table 6).

Multivariate analysis confirmed that PFS in first-line CT >12 months (HR 0.32; 95% CI 0.13–0.79; *p* = 0.01) was associated with better OS. On the other hand, no variables examined were associated with poor survival in multivariate analysis (Table 6, Figure 4).

As regards PFS, univariate analysis showed that sex (male vs. female) was negatively associated with PFS (5 vs. 7 months, *p* = 0.02). No variables were associated with better PFS in multivariate analysis (Table 7).

### 3.4. Safety

Hematological and non-hematological AEs are shown in (Table 8).

Overall, treatment was well tolerated, and most non-hematological toxicities were reported as G1 or 2 (e.g., asthenia in 67.3% of patients, diarrhea in 55.1%, arterial hypertension in 44.9%, and stomatitis in 34.7%). Hypertension was the most commonly reported G3–4 AE, affecting 9 out of 49 patients (18.4%). In addition, arterial or venous thromboembolism was observed in eight patients (16.3%), hemorrhagic events in five (10.2%), fistulas in two (4.1%), and proteinuria in four (8.2%). No cases of gastrointestinal perforation or severe heart failure were reported.

Neutropenia was the most frequently observed hematological AE with an incidence of 10.2%. Notably, three patients (6.1%) had infectious complications.

Maintenance therapy did not significantly increase toxicity since all of the G3–4 AEs occurred during induction therapy.

## 4. Discussion

The continuum of care for mCRC might include anti-angiogenic drug as anti-VEGF/VEGFR moAb and recombinant proteins in combination with fluoropyrimidine-based regimens in first- and second-line treatment, and multikinase inhibitors in refractory patients. Since the publication of the results of the VELOUR trial, aflibercept in combination with FOLFIRI has been widely used as second-line treatment in patients with mCRC. [12] However, outside the clinical trial framework, a few case series have explored the feasibility of aflibercept therapy in a non-favorably selected population (Table 1). The main evidence came from the Aflibercept Safety and health-related Quality-of-life Program (ASQoP) (number of patients, 798), prospective post-authorization safety OZONE study (*n* = 766), and two retrospective studies conducted by Ivanova et al. (*n* = 218) and Buchler et al. (*n* = 366) [13,14,15]. Furthermore, the clinical and molecular factors to select patients who are most likely to benefit from this treatment are largely unexplored.

Despite all the limitations of cross-trial comparison, our patient population was slightly older than that in the VELOUR study, with 40.8% of patients with ≥65 years compared with 36.1%, and was characterized by a higher tumor burden: multi-organ metastases in 73.5% of patients compared with 56.3% [26] (Table 2).

Although our patients were less-favorably selected than those enrolled in clinical trials, survival outcomes were consistent with the literature [12] (Table 1 and Table 5). In the pivotal trial, the benefit from aflibercept was observed across the prespecified subgroups, including patients who were exposed to bevacizumab in first-line treatment, and this subgroup accounted for only 30% of the overall population [9,13]. In contrast, in large case series, nearly half of patients with mCRC usually receive bevacizumab in combination with CT as first-line treatment (e.g., 46.2% in ASQoP, 58.6% in OZONE, etc.) [13,27] (Table 1), and this was in line with our observational data, with 79.6% of patients previously treated with bevacizumab (Table 3).

In our study, PFS in first-line therapy >12 months was the main factor associated with better OS in multivariate analysis (Table 6). In contrast, other factors, such as prior bevacizumab treatment, RAS and RAF mutational status, site of primary tumor, and hypertension, were not associated with survival (Table 6, Tables S1 and S2). Multi-organ involvement was associated with poor survival in univariate analysis: this was notably true for patients with bone metastases who had a median OS of 3 months (Table 6 and Figure S2). Although the number of patients was relatively small and results were not significant in multivariate analysis, this observation was consistent with other retrospective studies confirming bone metastases as a poor prognostic factor in mCRC. In fact, presence of bone metastases was usually associated with high tumor burden and reduced survival [28,29]. Notably, tumor burden being significantly related with survival was also suggested in the observational studies of Buchler et al. and Vera et al. [15,16].

To date, predictive factors of response to aflibercept are largely unknown. In a subgroup analysis from the VELOUR trial, a significantly greater treatment benefit from aflibercept was observed for patients with liver-only metastases, compared to those with multi-organ metastases or with other organs involved except the liver [26]. In addition, although the benefit from aflibercept was confirmed across all molecular subgroups, in a biomarker analysis from the VELOUR trial, a trend toward a deeper benefit was observed in RAS WT rather than in RAS-mutated CRC, with median OS of 16.0 months in the aflibercept group vs. 11.7 months in the placebo group for RAS WT patients (HR 0.7), and 12.6 vs. 11.2 months for RAS-mutated patients (HR 0.93) [30]. To date, only a retrospective observational study evaluated aflibercept-FOLFIRI as second-line treatment in a homogeneus series of 120 WT RAS CRC patients who had received a first-line standard CT plus anti-EGFR (epidermal growth factor receptor) moAb, describing an ORR of 33%, a median PFS of 6.9 months (95% CI: 6.1–7.8) and a median OS of 14.5 months (95% CI: 9.7–19.3) [27]. Unfortunately, our study could not address this crucial issue, due to sample limitation.

The safety profile of aflibercept in combination with FOLFIRI, before its marketing authorization, has been extensively studied in the ASQoP conducted by Riechelmann et al. [31] The patient population (*n* = 798) was approximately representative of the routine clinical practice. Nearly half of the patients had received bevacizumab in combination with CT in first-line treatment. Dose modifications and dose interruption of aflibercept due to AE occurred in 19% and 11.7%, respectively. Overall, 78% of patients experienced G3–4 AEs, including hypertension in 24.5%, neutropenia in 24.8%, and proteinuria in 3.6%. Fifteen treatment-related deaths (1.9%) were observed. However, a generally favorable patient’s perspective was reported in health-related quality of life scores.

As expected, the safety profile of aflibercept in our patient population was consistent with the VELOUR trial [9], ASQoP, and other case series (Table 1). Among the anti-VEGF class events, hypertension was the most commonly reported G3–4 AE both in our study and in VELOUR trial, affecting 18.4% and 19.3% of patients, respectively [12] (Table 8). Although cross-trial comparisons are not formally correct and may not provide definite conclusions, numerically lower rates of G3–4 hypertension were reported with the use of bevacizumab or ramucirumab in the TML, BEBYP, and RAISE trials (2%, 2%, and 11%, respectively) compared with those reported with aflibercept in the VELOUR trial and early access programs [9,12,32,33] (Table 1).

Notably, a higher number of potentially treatment-related any-grade thromboembolic events were observed in our case series (16.3%) as compared with those reported in the VELOUR trial (11.9%), ASQoP (8.6%), and other case series [12] (Table 1 and Table 8). These events deserve special attention and early management, as patients may experience long-term and potentially serious consequences. As previously reported in the literature, predictors of thromboembolic events in CRC included metastatic disease, number of comorbidities, and, more recently, the use of angiogenesis inhibitors [34,35]. In our study, the high rates of patients previously treated with bevacizumab, with multi-organ metastases and cardiovascular comorbidities might have influenced the incidence of thromboembolic events.

This study had several limitations, including the relatively small sample size, retrospective design, and lack of a control group that precluded a precise estimate of treatment benefit.

## 5. Conclusions

In conclusion, our study confirmed aflibercept-FOLFIRI as a feasible second-line treatment of mCRC in a patient population approximately representative of daily clinical practice, with PFS in first-line CT >12 months as the main predictor of better survival.

## Figures and Tables

**Table 1:** Summary of the main observational studies and early access programs of aflibercept in mCRC.

Ref.	Treatment Setting	Patients Previously Treated with Bevacizumab	N. Patients	Known Mutations	Prognostic Factors	ORR	DCR	PFS (Months)	OS (Months)	G3–4 Hypertension (%)	Thromboembolic Events (%)	Proteinuria G3–4 (%)	Dose Reduction Rate for Aflibercept Due to AEs (%)	Discontinuation Rate for Aflibercept Due to AEs (%)
Riechelmann et al. [12]	In combination with FOLFIRI after at least one prior oxaliplatin-based regimen.	46.2%	798	NA	NA	NA	NA	NA	NA	24.5%	8.6%	3.6%	19%	11.7%
Chau et al. [13]	In combination with FOLFIRI as second or further line of treatment.	58.6%	766	51.5% KRAS-mutated CRC 35.3% KRAS WT CRC 13.2% KRAS NA 2.7% BRAF-mutated CRC 22.5% BRAF WT CRC 74.8% BRAF NA	None	16.3%	50.8%	6.1	12.5	10.2%	NA	2.7%	36.2%	NA
Ivanova et al. [14]	In combination with FOLFIRI after at least one prior oxaliplatin-based regimen.	91.7%	218	43.7% RAS-mutated CRC 38.1% RAS WT CRC 17.9% RAS NA 3.6% BRAF-mutated CRC 17.3% BRAF WT CRC 79.1% BRAF NA	NA	21.1%	38.5%	3.7	9.6	6.4%	2.3%	NA	9.2%	23.4%
Buchler et al. [15]	Second-line treatment mainly in combination with FOLFIRI after standard CT plus bevacizumab.	100%	366	80.8% RAS-mutated CRC 12.3% RAS WT CRC 6.8% RAS NA 5.5% BRAF-mutated CRC 17.5% BRAF WT CRC 77.0% BRAF NA	PFS: duration of prior bevacizumab therapy. OS: presence of metastases at diagnosis.	NA	NA	5.6	14.2	NA	NA	NA	NA	2.3%
Vera et al. [16]	Second-line treatment in combination with FOLFIRI after standard CT plus anti-EGFR moAb.	0%	120	95% RAS/RAF WT CRC 5% BRAF-mutated CRC	OS: ECOG PS and number of metastatic sites.	33%	NA	6.9	14.5	7.5%	NA	NA	18.3%	6.7%
Feliu et al. [17]	Second-line treatment in combination with FOLFIRI.	60.6%	71	67.7% KRAS-mutated CRC 32.4% KRAS WT CRC	PFS: LDH, ECOG PS.	19.7%	57.7%	5.3	NA	11.3%	2.8%	1.4%	14.1%	9.9%
Yusof et al. [18]	Second-line treatment in combination with FOLFIRI.	8%	25	20.0% KRAS-mutated CRC 32.0% KRAS WT CRC 48.0% KRAS NA	NA	25%	62.5%	6.1	12.0	8%	NA	4%	NA	NA
Montes et al. [19]	Second-line treatment in combination with FOLFIRI.	47.4%	78	75.7% RAS-mutated CRC	PFS and OS: metachronous metastases, left-side primary tumor location.	21.8	70.5%	6.8	12.0	3.8%	NA	0%	NA	NA
Kim et al. [20]	Second-line treatment in combination with FOLFIRI.	96.2%	52	57.7% RAS/RAF-mutated CRC 36.5% RAS/RAF WT CRC 5.8% NA	PFS: response to first-line treatment, left-side primary tumor location, low baseline CEA level, WT RAS/RAF. OS: primary tumor location, baseline CEA level, RAS/RAF mutational status.	48.1%	84.6%	7.0	16.8	NA	NA	NA	NA	NA
Chong et al. [21]	Second-line treatment in combination with FOLFIRI.	15.8%	19	42.1% RAS-mutated CRC 52.6% RAS WT CRC 5.3% RAS NA 5.3% BRAF-mutated CRC 42.1% BRAF WT CRC 52.6% BRAF NA	NA	21.1%	42.1%	4.1	11.6	5.3%	5.3%	NA	NA	NA
Ottaiano et al (ARM B) [22]	Second-line treatment in combination with FOLFIRI.	100%	43	100% KRAS-mutated CRC	NA	11.6%	48.8%	NA	12.1	4.6%	0%	NA	2.3%	NA
Auvray et al. [23]	In combination with CT beyond second-line therapy.	86.2%	130	46.1% KRAS-mutated CRC 53.2% KRAS WT CRC 0.8% KRAS NA 3.07% NRAS-mutated CRC 46.1% NRAS CRC WT 50.8% NRAS NA 7.8% BRAF-mutated CRC 41.5% BRAF WT CRC 50.8% BRAF NA	PFS and OS: prior antiangiogenic treatment.	6.9%	45.4%	3.3	7.6	0.8%	0%	1.5%	NA	1.5%

Abbreviations: CRC, colorectal cancer; CT, chemotherapy; DCR, disease control rate; ECOG PS, Eastern Cooperative Oncology Group performance status; EGFR, epidermal growth factor receptor; G, grade; moAbs, monoclonal antibodies; N., number; NA, not assessed; ORR, overall response rate; OS, overall survival; PFS, progression-free survival; WT, wild-type.

**Table 2:** Characteristics of patients.

Baseline Characteristics	N. of Patients (*n* = 49)
Sex:	
Male	22 (44.9%)
Female	27 (55.1%)
Age:	
≥65	20 (40.8%)
<65	29 (59.2%)
ECOG PS:	
0	38 (77.6%)
1	11 (22.4%)
Primary tumor location:	
Left	33 (67.3%)
Right	16 (32.7%)
N. of metastatic organs involved at baseline:	
1	13 (26.5%)
>1	36 (73.5%)
Metastatic organs involved at baseline:	
Liver	36 (73.5%)
Peritoneum	13 (26.5%)
Bone	3 (6.1%)
Lymph nodes	24 (49.0%)
Lung	25 (51.0%)
Mucinous component:	
Yes	18 (36.7%)
No	31 (63.3%)
RAS, RAF, MSI status:	
KRAS-mutated	34 (69.4%)
NRAS-mutated	3 (6.1%)
BRAF-mutated among patients tested (*n* = 41)	2 out of 41 (4.9%)
MSI-H among patients tested (*n* = 18)	2 out of 18 (11.1%)
Weight loss in last 3 months:	
≥5 kg	44 (89.8%)
<5 kg	5 (10.2%)

Abbreviations: CRC, colorectal cancer; ECOG PS, Eastern Cooperative Oncology Group performance status; MSI, microsatellite instability; N, number.

**Table 3:** Characteristics of patients concerning prior therapies.

Characteristic	n° (%)
Previous surgery:	
Yes	34 (69.4%)
No	15 (30.6%)
Prior adjuvant CT:	
Yes	9 (18.4%)
No	40 (81.6%)
Best response to first-line CT:	
CR	5 (10.2%)
PR	20 (40.8%)
SD	12 (24.5%)
PD	12 (24.5%)
PFS in first-line CT:	
>6 months	38 (77.6%)
>12 months	14 (35.9%)
Prior bevacizumab:	
Yes	39 (79.6%)
No	10 (20.4%)

Abbreviations: CR, complete response; CT, chemotherapy; PD, progressive disease; PFS, progression-free survival; WT, wild-type; PR, partial response; SD, stable disease.

**Table 4:** Prior cardiovascular comorbidities.

Characteristic	n° (percentage)
Prior hypertension:	
Yes	16 (32.6%)
No	33 (67.3%)
Prior thromboembolic events:	
Yes	3 (6.1%)
No	46 (93.9%)
Prior hemorrhagic events:	
Yes	3 (6.1%)
No	46 (93.9%)

**Table 5:** Best response, PFS, OS, and treatment data.

Characteristic	All Patients (*n* = 49) N° (%)
CR	2 (4.1%)
PR	4 (8.2%)
SD	18 (36.7%)
PD	22 (44.9%)
ORR	6 (12.3%)
DCR	24 (49.1%)
NA	3 (6.1%)
PFS M-months (95% CI)	6 (5–7)
OS M-months (95% CI)	13 (10–18)
Cycles	
Median	7
Range	1–13
>10	17 (43.7%)
Dose reduction	CT: 35 (71.4%) Aflibercept: 13 (26.5%)
Treatment Interruption (aflibercept, FOLFIRI or both) due to AEs	20 (40.8%)
Further Lines of treatment	31 (63.3%)
TAS-102	10
Regorafenib	10
Other	11

Abbreviations: AE, adverse event; CR, complete response; CT, chemotherapy; DCR, disease control rate; ECOG PS, Eastern Cooperative Oncology Group performance status; M, median; N, number; NA, not assessed; ORR, overall response rate; OS, overall survival; PD, progressive disease; PR, partial response; PFS, progression-free survival; SD, stable disease.

**Table 6:** Univariate and multivariate analysis for OS (N. of patients = 49).

Characteristic	Univariate			Multivariate		
	HR	CI 95%	*p*	HR	CI 95%	*p*
ECOG PS (1 vs. 0)	2.31	1.01–5.28	0.03	1.32	0.47–3.74	0.6
Sex (male vs. female)	1.30	0.68–2.48	0.4			
Primary site (right vs. left)	1.43	0.71–2.89	0.3			
N. of metastatic sites > 1	2.27	1.03–5.03	0.03	2.18	0.67–7.03	0.2
Liver metastases (yes vs. no)	2.31	1.01–5.28	0.03	0.79	0.23–2.73	0.7
Bone metastases (yes vs. no)	7.00	1.50–32.66	<0.01	3.52	0.64–19.31	0.1
Lung metastases (yes vs. no)	1.60	0.80–3.20	0.2			
KRAS WT (yes vs. no)	0.85	0.40–1.80	0.7			
NRAS WT (yes vs. no)	1.39	0.33–5.81	0.6			
BRAF WT (yes vs. no)	0.47	0.10–2.05	0.3			
Mucinous component (yes vs. no)	0.87	0.44–1.74	0.7			
Weight loss in last 3 months (no vs. yes)	1.15	0.41–3.28	0.7			
Prior bevacizumab (yes vs. no)	1.25	0.55–2.86	0.5			
Prior adjuvant CT (yes vs. no)	0.57	0.22–1.47	0.2			
PFS in first-line CT > 6 months (yes vs. no)	0.66	0.30–1.41	0.3			
PFS in first-line CT > 12 months (yes vs. no)	0.33	0.15–0.72	<0.01	0.32	0.13–0.79	0.01
Maintenance (yes vs. no)	0.44	0.21–0.89	0.02	0.73	0.32–1.65	0.5
Cycles of therapy > 10	0.50	0.25–1.00	0.04	0.50	0.22–1.16	0.1
Age > 65 (yes vs. no)	1.28	0.67–2.45	0.5			
Neutropenia G3–4 (yes vs. no)	0.84	0.29–2.41	0.7			
Diarrhea G3–4 (yes vs. no)	1.95	0.72–5.25	0.2			
Hypertension G1/2 (yes vs. no)	1.20	0.61–2.35	0.6			
Hypertension G3–4 (yes vs. no)	0.65	0.28–1.50	0.3			

Abbreviations: CT, chemotherapy; ECOG PS, Eastern Cooperative Oncology Group performance status; G, grade; N, number; OS, overall survival; PFS, progression-free survival; WT, wild-type.

**Table 7:** Univariate analysis for PFS (N. of patients = 49).

Characteristic	HR	CI 95%	*p*
ECOG PS (1 vs. 0)	1.75	0.80–3.80	0.1
Sex (male vs. female)	1.97	1.07–3.64	0.02
Site (right vs. left)	0.99	0.52–1.90	0.9
N. of metastatic sites>1	1.48	0.77–2.85	0.2
Liver metastases (yes vs. no)	1.70	0.88–3.28	0.1
Bone metastases (yes vs. no)	3.73	0.81–17.07	0.1
Lung metastases (yes vs. no)	0.78	0.43–1.41	0.4
KRAS WT (yes vs. no)	1.27	0.66–2.44	0.5
NRAS WT (yes vs. no)	0.47	0.14–1.55	0.2
BRAF WT (yes vs. no)	1.30	0.30–5.48	0.7
Mucinous component (yes vs. no)	0.60	0.32–1.14	0.1
Weight loss in last 3 months (no vs. yes)	0.82	0.32–2.12	0.7
Prior bevacizumab (yes vs. no)	1.44	0.71–2.94	0.3
Prior adjuvant CT (yes vs. no)	0.85	0.39–1.33	0.7
PFS in first-line CT > 6 months (yes vs. no)	0.85	0.42–1.73	0.6
PFS in first-line CT > 12 months (yes vs. no)	0.76	0.40–1.48	0.4
Age > 65 (yes vs. no)	1.42	0.76–2,73	0.3
Neutropenia G1–2 (yes vs. no)	1.15	0.57–2.32	0.7
Neutropenia G3–4 (yes vs. no)	0.90	0.32–2.57	0.8
Diarrhea G1/2 (yes vs. no)	0.54	0.29–1.02	0.06
Diarrhea G3/4 (yes vs. no)	0.97	0.38–2.49	0.9
Hypertension G1/2 (yes vs. no)	0.68	0.47–1.26	0.2
Hypertension G3/4 (yes vs. no)	0.47	0.21–1.07	0.07

Abbreviations: CT, chemotherapy; ECOG PS, Eastern Cooperative Oncology Group performance status; G, grade; N, number; PFS, progression-free survival; WT, wild-type.

**Table 8:** Most relevant adverse events (AEs) and grade.

AE	N. of Patients (*n* = 49)
Leukopenia G1/2	13 (26.5%)
Leukopenia G3/4	2 (4.1%)
Neutropenia G1/2	12 (24.5%)
Neutropenia G3/4	5 (10.2%)
Platelet count decreased G1/2	3 (6.1%)
Platelet count decreased G3/4	1 (2.0%)
Diarrhea G1/2	27 (55.1%)
Diarrhea G3/4	5 (10.2%)
Proteinuria	4 (8.2%)
Asthenia G1/2	33 (67.3%)
Asthenia G3	7 (14.3%)
Arterial hypertension G1/2	22 (44.9%)
Arterial hypertension G3/4	9 (18.4%)
Hypertransaminasemia G1/2	8 (16.3%)
Hypertransaminasemia G3/4	1 (2.0%)
Stomatitis G1/2	17 (34.7%)
Stomatitis G3/4	3 (6.1%)
Infectious complications	3 (6.1%)
Hypersensitivity	2 (4.1%)
Arterial/venous thromboembolism	8 (16.3%)
Perforation	0
Hemorrhagic events	5 (10.2%)
Fistulas	2 (4.1%)
Heart failure	0

Abbreviations: AE, adverse event; G, grade; N, number.

## Data Availability

Data available on request to the corresponding author.

## Supplementary Materials

The following are available online at https://www.mdpi.com/article/10.3390/cancers13153863/s1. Figure S1. Overall Survival of FOLFIRI + aflibercept according to ECOG, Figure S2. Overall Survival of FOLFIRI + aflibercept according to presence of bone metastases, Table S1. Treatment response according to KRAS status, Table S2. Treatment response according to primary site.

## Abbreviations

AE: adverse event; ASQoP: Aflibercept Safety and health-related Quality-of-life Program; CR: complete response; CRC: colorectal cancer; CT: chemotherapy; DCR: disease control rate; ECOG PS: Eastern Cooperative Oncology Group performance status; EGFR: epidermal growth factor receptor; FDA: Food and Drug Administration; G: grade; mCRC: metastatic colorectal cancer; moAbs: monoclonal antibodies; MSI: microsatellite instability; ORR: overall response rate; OS: overall survival; PFS: progression-free survival; PIGF: placental growth factor; PR: partial response; SD: stable disease; VEGF: vascular endothelial growth factor; VEGFR: vascular endothelial growth factor receptor.
