# Supplementary material for: Aflibercept Plus FOLFIRI as Second-Line Treatment for Metastatic Colorectal Cancer: A Single-Institution Real-Life Experience
Source: Cancers (Basel). 2021 Jul 31;13(15):3863. doi: 10.3390/cancers13153863 (PMC8345481; doi:10.3390/cancers13153863)
Supplement: Supplementary file 1 [file cancers-13-03863-s001.zip › cancers-1309364-supplementary.pdf]

cancers

Supplementary Materials: Aflibercept plus FOLFIRI as Second-Line Treatment for Metastatic Colorectal Cancer: A Single-Institution Real-Life Experience

Daniele Lavacchi, Giandomenico Roviello, Elisa Giommoni, Lorenzo Dreoni, Silvia Derio, Marco Brugia, Amedeo Amedei, Serena Pillozzi and Lorenzo Antonuzzo

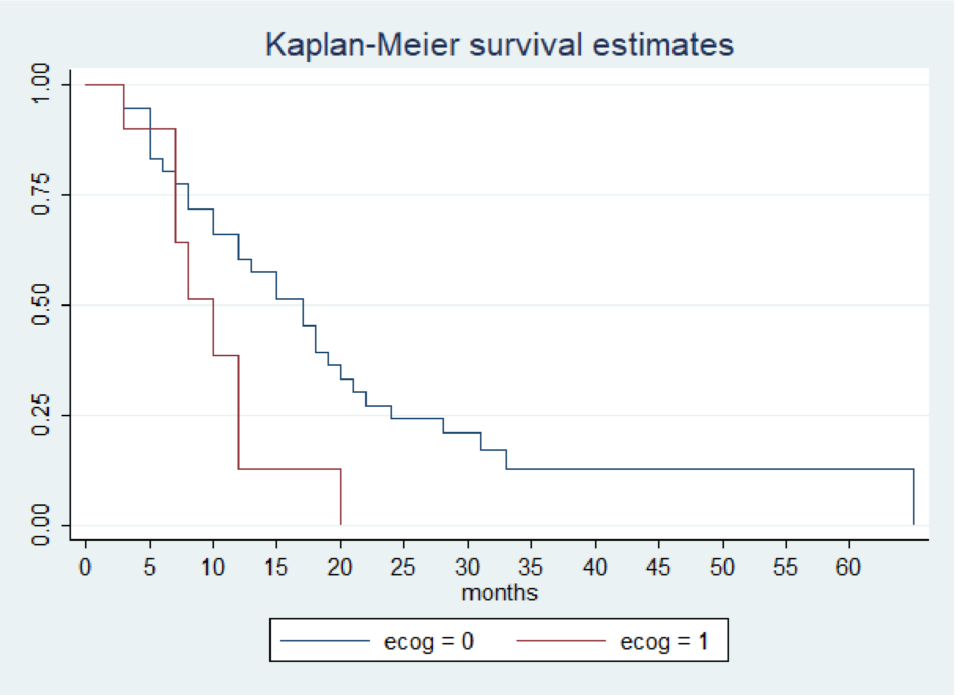

Figure S1. Overall Survival of FOLFIRI+aflibercept according to ECOG.

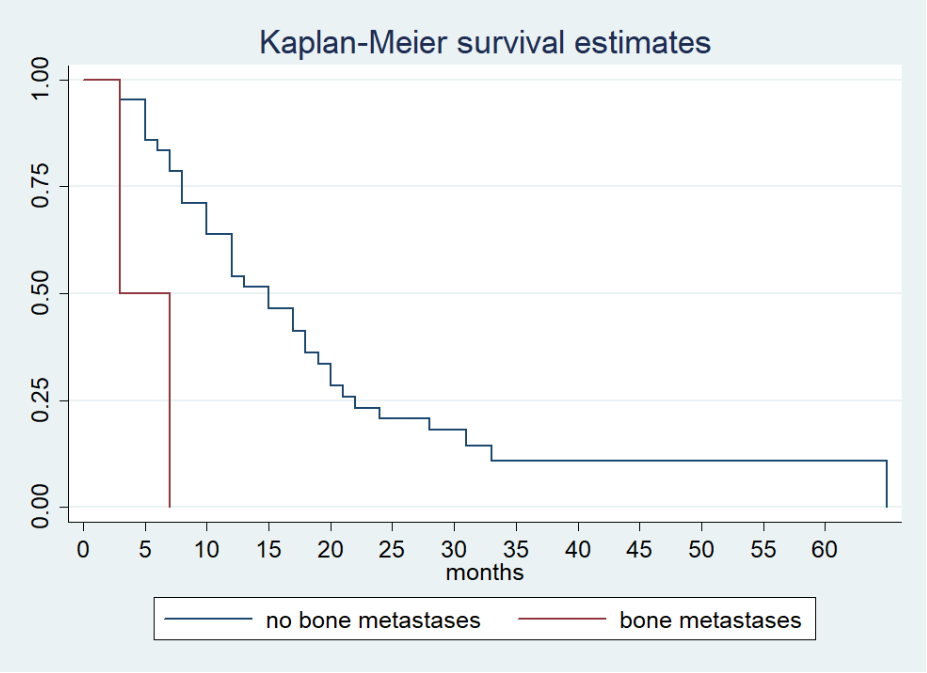

Figure S2. Overall Survival of FOLFIRI+aflibercept according to presence of bone metastases.

Table S1. Treatment response according to KRAS status.

**Title**	**KRAS-Mutated CRC** ***n*** **= 32**	**WT KRAS CRC** ***n*** **= 13**
CR	2 (6.2%)	0 (0%)
PR	0 (0%)	3 (23.1%)
SD	14 (43.7%)	4 (30.8%)
PD	16 (50%)	6 (46.1%)

Abbreviations: CR: complete response; PD: progressive disease; PR: partial response; SD: stable disease.

Table S2. Treatment response according to primary site.

**Title**	**Left-sided CRC** ***n*** **= 32**	**Right-sided CRC** ***n*** **= 14**
CR	2 (6.2%)	0 (0%)
PR	3 (9.4%)	1 (7.1%)
SD	13 (40.6%)	5 (35.7%)
PD	14 (63.6%)	8 (57.1%)

Abbreviations: CR: complete response; PD: progressive disease; PR: partial response; SD: stable disease.
